# Graphene Metamaterials for Intense, Tunable, and Compact Extreme Ultraviolet and X‐Ray Sources

**DOI:** 10.1002/advs.201901609

**Published:** 2019-10-02

**Authors:** Andrea Pizzi, Gilles Rosolen, Liang Jie Wong, Rasmus Ischebeck, Marin Soljačić, Thomas Feurer, Ido Kaminer

**Affiliations:** ^1^ Cavendish Laboratory University of Cambridge Cambridge CB3 0HE UK; ^2^ Micro and Nanophotonic Materials Group Research Institute for Materials Science and Engineering University of Mons Place du Parc 20 7000 Mons Belgium; ^3^ School of Electrical and Electronic Engineering Nanyang Technological University 50 Nanyang Ave Singapore 639798 Singapore; ^4^ Singapore Institute of Manufacturing Technology 2 Fusionopolis Way, Innovis Singapore 138634 Singapore; ^5^ Paul Scherrer Institute 5232 Villigen Switzerland; ^6^ Department of Physics Massachusetts Institute of Technology 77 Massachusetts Avenue Cambridge MA 02139 USA; ^7^ Institute of Applied Physics University of Bern Bern 3012 Switzerland; ^8^ Department of Electrical Engineering Technion – Israel Institute of Technology Haifa 32000 Israel

**Keywords:** free‐electrons, graphene, metamaterials, nanophotonics, plasmons, X‐ray sources

## Abstract

The interaction of electrons with strong electromagnetic fields is fundamental to the ability to design high‐quality radiation sources. At the core of all such sources is a tradeoff between compactness and higher output radiation intensities. Conventional photonic devices are limited in size by their operating wavelength, which helps compactness at the cost of a small interaction area. Here, plasmonic modes supported by multilayer graphene metamaterials are shown to provide a larger interaction area with the electron beam, while also tapping into the extreme confinement of graphene plasmons to generate high‐frequency photons with relatively low‐energy electrons available from tabletop sources. For 5 MeV electrons, a metamaterial of 50 layers and length 50 µm, and a beam current of 1.7 µA, it is, for instance, possible to generate X‐rays of intensity 1.5 × 10^7^ photons sr^−1^ s^−1^ 1%BW, 580 times more than for a single‐layer design. The frequency of the driving laser dynamically tunes the photon emission spectrum. This work demonstrates a unique free‐electron light source, wherein the electron mean free path in a given material is longer than the device length, relaxing the requirements of complex electron beam systems and potentially paving the way to high‐yield, compact, and tunable X‐ray sources.

## Graphical abstract

The short operating wavelength of nanophotonic devices is crucial for compactness but also typically limits the interaction area and hence yield. Graphene metamaterials can enormously scale up the interaction area of plasmon‐based free‐electron light sources, paving the way to high‐yield, compact, and tunable sources of extreme ultraviolet to gamma rays.

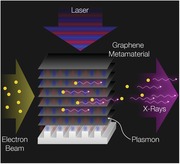

## 1 Introduction

In the last decade, growing interest in highly integrated light sources has led to various designs for compact free‐electron radiation emitters, which include dielectric‐based undulators,^1^ light wells,^2^, ^3^ and plasmonic undulators based on graphene^4^ and metallic nanogratings.^5^ Together with the progress of highly integrated electron accelerators^6^, ^7^, ^8^ and narrow‐linewidth miniaturized lasers,^9^ the development of nanoscale undulators paves the way toward the realization of on‐chip extreme ultraviolet (EUV) and X‐ray light sources, with promising applications in medicine, engineering, and natural sciences.^10^, ^11^, ^12^, ^13^ Graphene plasmons have been shown to be especially suitable for the manipulation of light–matter interaction,^14^ owing to their dynamic tunability, low losses, and strong confinement.^15^, ^16^, ^17^, ^18^, ^19^, ^20^, ^21^ In particular, the highly confined plasmons possess very high momentum and scatter off into highly directional X‐rays upon interaction with just moderately relativistic free electrons.^4^ However, the strong field confinement of such plasmons implies a small transverse extent of the polaritonic field, which limits the number of interacting electrons and hence the emitted X‐ray intensity. This type of limitation is experienced by nanophotonic technologies in general, since the physical area for light–matter interaction is typically on the order of the micro/nanoscale photonic wavelengths.

Here, we present a concept in which graphene metamaterials are used to increase the electron–plasmon interaction area, thereby scaling up the output radiation intensity by a factor as much as 580 with respect to the single graphene layer setup. Prior studies have shown the ability of graphene *multilayer* structures to support metamaterial resonant plasmons (MRPs),^21^, ^22^, ^23^, ^24^, ^25^, ^26^, ^27^, ^28^, ^29^, ^30^, ^31^, ^32^ and we propose to use them to enhance the output intensity in free‐electron‐based light sources. Layered conducting structures have also been considered in other contexts like the generation of visible Cherenkov radiation.^33^ By quantitatively calculating all competing scattering processes in such structures, we show that for the first time it becomes possible to reach significant radiation yield by electrons moving *through* a metamaterial, without a need of complicated beam control to guide the electron beam through vacuum. We show that graphene multilayers enable significant improvement of the radiation output intensities by allowing larger electron beams—and hence more electrons per pulse—to fit into the device. We find that, depending mostly on the graphene quality (i.e., conductivity), there exists an optimum number of layers in the metamaterial that maximizes the output radiation intensity. We also show that the variety of modes resonating in the metamaterial at different driving laser frequencies enables us to generate multiple X‐ray harmonics (for instance, from 2.7 to 12 keV photons using 5 MeV electrons), making our concept useful for applications like time‐resolved X‐ray spectroscopy for ultrafast imaging of electronic‐state transitions and chemical reactions.^34^, ^35^

## 2 Results

### 2.1 Intense Free‐Electron Light Sources Based on Graphene Metamaterials

**Figure 1:**
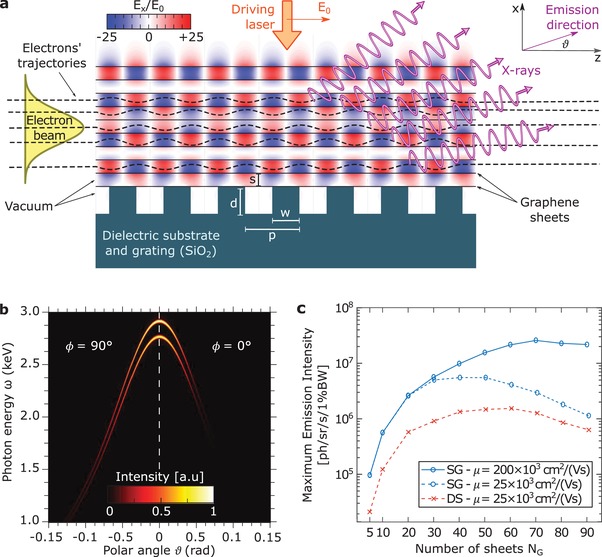
Graphene metamaterial plasmon‐driven free‐electron source of short‐wavelength light. a) 2D schematic of the setup. The driving laser excites MRPs by means of a grating structure; the free electrons in the beam (with trajectories as dashed black lines) oscillate due to the MRP field (*E_x_* in red and blue), emitting EUV to X‐ray radiation. b) The emission is shown to be highly directional and nearly monochromatic by plotting the emitted radiation intensity in arbitrary units (for *N*_G_ = 50 graphene layers, *N*_P_ = 100 grating periods and at driving laser wavelength of λ_0_ = 7.086 µm). c) We show how the emission intensity can be scaled up by increasing the number *N*_G_ of graphene sheets in the metamaterial, increasing the interaction area, both in the case of suspended graphene (SG) and dielectric spacing (DS). Depending mainly on the mobility *µ* of the electrons in the graphene, we find an optimum *N*_G_^*^ at which the output intensity is maximized (for *N*_P_ = 500). We consider a maximum electric field magnitude in the structure *E*_max_ = 3 GV m^−1^, grating periodicity *p* = 100 nm, groove depth *d* = 50 nm, grooves' width *w* = 50 nm, dielectric spacings' thickness *s* = 25 nm.

We present plasmon‐driven free‐electron light sources based on multilayer graphene metamaterials that allow highly directional EUV and X‐ray light sources. This section shows how the metamaterial design can enhance the emission intensity by factors > 500 relative to a single‐layer device. The schematic of the setup is shown in **Figure** **1**a. While our theory is completely general, we choose the following parameters to exemplify the concept. Consider a periodic grating of period *p* = 100 nm, groove depth *d* = 50 nm, and width *w* = 50 nm on the top of a silica substrate, all placed in a vacuum environment. On top of the grating we place an array of *N*_G_ suspended graphene sheets, with *s* = 25 nm being the distance between two consecutive sheets. The array of suspended graphene layers could be potentially realized exploiting a microfabricated dielectric scaffold that holds up the layers by their edges. In analogy with realizations of suspended single layers,^36^, ^37^ such a scaffold may be realized exploiting lithographic and etching techniques. An alternative realization of the device could exploit silica spacing layers between consecutive graphene sheets to increase the structure mechanical stability, at the price of a larger electron–matter interaction and bremsstrahlung background radiation, but still allowing substantial radiation from electron–plasmon interaction (details in Section S1, Supporting Information). The Fermi energy of the graphene layers may be tuned via electrical gating or chemical doping.^38^, ^39^ A driving laser of wavelength λ_0_ is incident from the top of the structure, normal to the graphene sheets and polarized with the electric field parallel to the periodicity direction (i.e., *z*). The laser couples to MRPs through the grating.^40^, ^41^ The driving laser electric field strength *E*_0_ is tuned in such a way that the maximum electric field magnitude on the graphene sheets is *E*_max_ = 3 GV m^−1^.^42^ For instance, for *N*_G_ = 50 we find *E*_0_ = 264 MV m^−1^, which for a realistic waist radius *w*_0_ = 10 µm necessitates a driving laser power *πE*_0_^2^ *w*_0_^2^/*Z*_0_ = 58 kW, *Z*_0_ being the vacuum impedance.^43^ Initially, the electrons have a kinetic energy of 5 MeV, negligible energy spread, and a gaussian distribution in position with standard transverse deviations *σ_x_* = *σ_y_* = σ. The average electron current is *I*_C_, directed parallel to *z*, and aligned at the center of the multilayer graphene stack (i.e., with axis placed at a distance *x*_0_ = (*N*_G_ − 1)*s*/2 above the grating). The beam properties are chosen such that only a negligible fraction of the electrons hits the substrate and generate background bremsstrahlung from it.^44^ The electrons travel through a structure of length *L* and interact with the MRPs resulting in the emission of high‐energy photons (the device working principle is shown with pictorial animations in Video S1, Supporting Information).

By means of ab initio simulations, we predict intense, nearly monochromatic, and directional output radiation. The simulations consist of three main steps: (1) exact electromagnetic field solution in the graphene metamaterial, including the excitation of MRPs by the incident field; (2) numerical solution of the Newton–Lorentz equation of motion to find the electrons' trajectories in the field; (3) computation of the emitted radiation by the Liénard–Wiechert potentials.^45^ Further details are provided in the Methods. In Figure 1b, we show the emitted radiation spectral intensity $\frac{d^{2}I}{d\omega d\Omega }$ as a function of the polar angle θ at two fixed azimuthal angles ϕ = 0° (right) and ϕ = 90° (left). We tune the driving laser wavelength to excite a MRP. The field profile of such a MRP is analogous to the one of Figure 1a, just extended to a thicker stack, and its longitudinal (i.e., parallel to *z*) component of the plasmon wavevector is *q* = 2π/*p*. Throughout the entire stack, the electrons interact both with the propagating and the backward propagating (with respect to *z*) components of the standing plasmonic field resulting in a pair of spectral emission lines per plasmon mode. The central emission energy depends on the observation direction and, at a given direction (which can be selected with a shutter), the emission bandwidth is extremely narrow (comparable with that of X‐ray free electron lasers),^46^ a feature we refer to using the term “nearly monochromatic.” The maximal emission energy and intensity are reached in the forward direction, where the line bandwidth amounts to 0.86% (for the parameters in Figure 1, with *L* = 10 µm). More intense emission lines and narrower bandwidths are obtained for longer structures. For instance, for *L* = 50 µm the bandwidth amounts to 0.17% and the intensity to 1.5 × 10^7^ photons sr^−1^ s^−1^ 1%BW, 580 times larger than the intensity 2.4 × 10^3^ photons sr^−1^ s^−1^ 1%BW obtained in a single‐layer setup with *x*_0_ = 30 nm and σ = 10 nm. Note that, in our regime of interest, the emission bandwidth is set mostly by the (finite) structure length and scales as ≈1/*L*.^4^

We demonstrate how the output radiation can be scaled up by increasing the number of layers in the stack. On the one hand, thanks to their ability to support high‐intensity lobes over a wide area in the transverse direction, graphene metamaterials allow electron beams of large transversal size, and thus current, to fit into the device. On the other hand, due to losses in the graphene, the power of the driving laser cannot penetrate uniformly throughout the entire metamaterial at large *N*_G_. Therefore, choosing values of *N*_G_ that are too large results in the MRP electric field being concentrated in the upper part of the metamaterial and leads to a reduction of the interaction area. The competition between these two effects gives rise to an optimum *N*_G_^*^ at which the output intensity is maximized. In Figure 1c, we show how the maximum emission intensity *I*_max_ (reached in the forward direction θ = 0°) scales with *N*_G_. To demonstrate the influence of the losses on this limit, we perform simulations for different values of the mobility *µ* of electrons in graphene, for instance, finding for the suspended graphene *N*_G_^*^ = 40, 70 for *µ* = 25, 200 × 10^3^ cm^2^ V^−1^ s^−1^, respectively. This result highlights that a higher electron mobility (i.e., lower graphene losses) leads to a higher value of the optimum *N*_G_^*^ and of the emission intensity. Graphene is therefore an ideal candidate for the realization of the metamaterial, combining strong MRP confinement and tunability with small losses.

### 2.2 Higher Harmonics Radiation from Graphene Metamaterials

The different MRPs supported by graphene metamaterials scatter off into different emitted photon energies for a fixed electron energy and fixed grating periodicity, and their relative amplitudes and phases can be control via the MRP excitation and metamaterial design. For instance, we show that, for 5 MeV electrons and a grating periodicity *p* = 100 nm, multiple output photon energies at 5.5, 8.5, and 11.5 keV (and beyond) can be obtained in the forward (θ = 0°) direction. The underlying reason for this possibility is that the field profile of the MRP is not perfectly sinusoidal and is rather composed of a sum of different spatial harmonic modes. In the setup presented here, the monochromatic driving laser in general excites all these modes, but one mode is typically dominant with respect to the others and we define it as the *order* of the MRP. Tuning the driving laser frequency allows one to choose which order of MRP to excite (corresponding to different absorption peaks in Figure 3) in our free‐electron plasmonic undulator. We label each MRP order with a pair of integers *n* = 1, 2, 3, … and *m* = 0, 1, …, *N*_G_ − 1, which enumerate the possible plasmon longitudinal and transverse momenta, respectively (see Section S2 and Figure S3, Supporting Information, for further details). More precisely, *n* corresponds to the number of spatial periods of the MRP per grating period *p* in the *z* direction. Each of these orders results in a pair of emission lines at frequencies ω_±_ given by^4^

(1) $$\omega_{\pm }=\frac{n\beta \omega_{p}\pm \omega_{0}}{1-\beta \cos\theta }$$

where β = *v*/*c* is the reduced electron velocity, ω_0_ = 2*πc*/λ_0_ is the angular frequency of the driving laser, and ω_p_ = 2*πc*/*p* is the effective frequency defined by the spatial periodicity *p* along *z*. Generally, a higher MRP order *n* leads to a shorter periodicity of the field in the *z*‐direction, which yields to a higher frequency of oscillation of the free electrons, and consequently higher energy of the emitted radiation (this is illustrated with a pictorial animation for *n* = 2 in Video S1, Supporting Information). Notice that, according to Equation (1), the output photon energy is not directly affected by the laser power. In fact, a higher laser power with all other parameters held constant simply leads to a higher output intensity.

**Figure 2:**
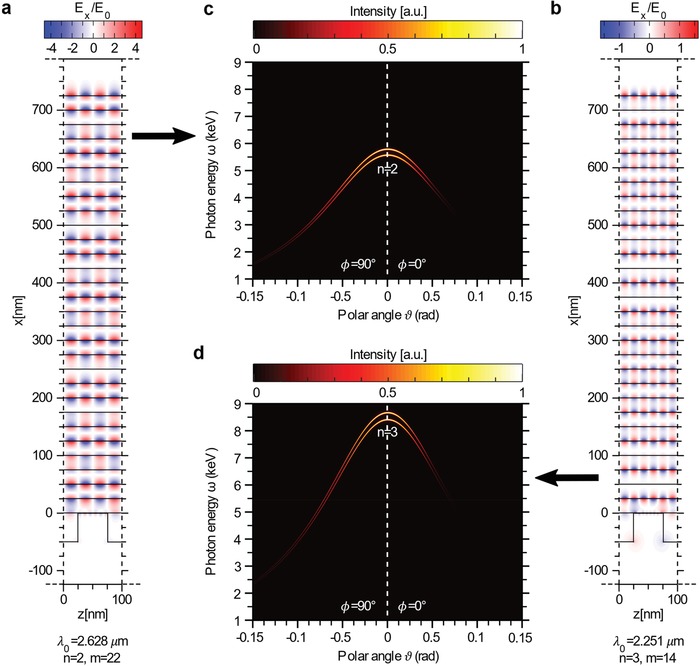
Tunable radiation emission: higher orders (*n*) MRPs. The MRPs are shown plotting in red and blue the transverse component of the electric field for MRPs with a) *n* = 2, *m* = 22, λ_0_ = 2.628 µm, *E*_0_ = 329 MV m^−1^ and b) *n* = 3, *m* = 14, λ_0_ = 2.251 µm, *E*_0_ = 810 MV m^−1^. c,d) The corresponding emitted radiation spectra are shown, respectively, for an electron beam with the characteristics specified in ref. 44. In each structure, a different *n*th emission harmonic is brightest: c) the second harmonic for structure (a), and d) the third harmonic for structure (b). In these simulations, we consider *N*_G_ = 30 graphene sheets and *N*_P_ = 50 grating periods (total length is *L* = 5 µm).

In **Figure** **2**, we show two examples of the field distributions and respective emission spectra for *n* = 2, *m* = 22 (Figure 2a,c) and for *n* = 3, *m* = 14 (Figure 2b,d). In Figure 2c,d, we observe that the brighter pair of emission lines is the one belonging to the *n*th harmonics (the other harmonics being much less intense and visible only with a logarithmic colorbar). For a longer structure of *L* = 50 µm (vs *L* = 5 µm in Figure 2), the emission lines are ten times narrower and the maximum emission intensity amounts to 1.1 × 10^6^ and 1.4 × 10^5^ photons sr^−1^ s^−1^ 1%BW for *n* = 2 and *n* = 3, respectively.

We now study the tunability of the radiation emission that is offered by graphene metamaterial structures. As shown in Figure 2c,d, the emitted radiation spectrum depends on the mode decomposition of the MRP along the beam axis, that is, on the order *n*. Conversely, the MRP wavevector component transversal to the beam axis (related to the order *m*) does not alter the central position of a pair of emission lines, which is obtained from Equation (1) to be ω_c_ = *nβω*_p_/(1 − βcosθ). Nevertheless, MRPs that have different *m* are excited at different driving laser frequencies ω_0_, and thus *m* affects the *distance* between the spectral emission lines of a pair, that is, *δω* =  2ω_0_/(1 − βcosθ). This way, the driving laser frequency controls both the central frequency of the radiation emission and the fine features of the emission spectrum.

The excitation of a single plasmonic mode results in a nearly monochromatic emission in a given direction. Nonetheless, coupling to several modes simultaneously with a large bandwidth driving laser opens exciting possibilities. For instance, coupling to several modes with different *n* leads to different pairs of bright emission lines at the corresponding harmonic values *n*, whereas coupling to several different *m* and same *n* leads to several pairs of bright emission lines surrounding the *n*th harmonic. Importantly, the EUV or X‐ray emission for a single electron (or for a properly microbunched electron beam, which we do not consider here) will maintain *coherence* between the different spectral peaks (as long as the driving laser preserves coherence between the different resonant wavelength, which is often the case in femtosecond pulses). Having coherence in EUV and X‐rays means that the emission could be shaped in time, for example, creating multiple narrow pulse peaks in time, or even a comb (if there are enough harmonic orders *n*). All these temporal features will be on *zeptosecond timescales*. It is a unique and intriguing opportunity opened by the MRPs—enabling to translate the coherence of the excitation pulse from a relatively narrow range of wavelengths in the IR, to a much wider spectral range in short‐wavelength radiation.

**Figure 3:**
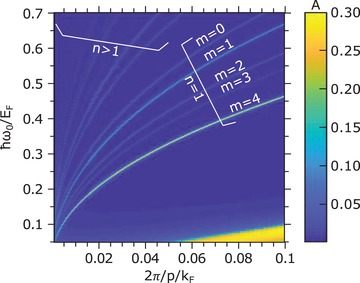
Mode dispersion of the graphene multilayer structure showing the MRPs numbered by the integers *n*, *m*. The map shows the absorption *A* computed and plotted within the nonlocal random phase approximation for the graphene conductivity σ_g_ = σ_g_(ω_0_, *q* = 2π/*p*) as a function of the normalized inverse periodicity and frequency. For *n* = 1, we find multiple absorption peaks that correspond to different MRPs and that we enumerate with an integer *m*. Other higher energy absorption lines correspond instead to MRPs of order *n* > 1 that is possessing larger longitudinal momenta. Note that here the electron mobility has been artificially reduced to increase the visibility of the absorption lines that would otherwise (for higher values of *µ*) be even narrower. We choose electron mobility *µ* = 10 × 10^3^ cm^2^ V^−1^ s^−1^, denote the Fermi wavevector with *k*_F_, and consider *N*_G_ = 5 graphene layers with spacing *s* = *p*/4. Note that since the longitudinal momentum for *n*th order MRPs is *q* = 2*πn*/*p*, we expect stronger nonlocal effects to occur for *n* > 1 (the effects of nonlocalities here are nevertheless negligible and are addressed more in detail in Section S4, Supporting Information).

The emission spectrum is broadened by the finite interaction length, depending on the length of the structure and electron beam parameters. Typical parameters will result in having the spectral peaks of different values of *m* all overlap. When simultaneously coupling to several MRPs with same order *n* but different order *m*, the total spectrum then looks similar to the case of exciting only a single mode, eventually with broader spectral emission peaks due to the imperfect overlapping of the emission lines associated to the various values of *m* (see 3, Supporting Information). For the purpose of shaping the output spectrum and thus also the output temporal pulse features, we study the relative output radiation intensity from each mode. We notice that *m* influences the efficiency of the coupling of the driving laser with the MRPs, since each of them has a different coupling efficiency and a different quality factor (**Figure** **3**). Nonetheless, it is possible to tune the input driving laser electric field strength *E*_0_ to reach the same maximum field *E*_max_ in the structure for each *m*, so that the output radiation intensity does not significantly depend on the choice of *m*.

## 3 Discussion

We showed the ability of graphene metamaterials to scale up the emitted intensity of plasmonic‐driven free‐electron light sources by factors >500, with the potential to achieve even greater intensities for a larger electron mobility. Our setup with *N*_G_ > 1 exhibits strong MRP fields also at distances of several “plasmon decay constants” λ_d_ = 2π/*q* = *p*/*n* (Figures 1a and 2a,b). This overcomes the inherent limit characterizing single‐layer scenarios, where an increase of the output photon energy by means of a reduction of *p* comes at the price of a reduced physical area for electron interaction with the polariton field.^47^, ^48^ Our setup enables the use of large, high current circular beams, reducing the complexity of the beam preparation and facilitating more intense X‐ray output.

Graphene metamaterials provide us for the first time a system for high‐quality EUV and X‐ray generation that works with electrons that *penetrate through* the device, instead of moving through vacuum, since their mean free path is longer than the required device length. Such devices could open new opportunities in designs of future radiation sources, reducing constrains that are related to vacuum considerations and to the quality of the electron beam. Of course, there is a trade‐off with secondary emission and damage, which poses strict limits on usable electron energies. To explore these limitations, we also study designs in which dielectric materials are used as spacers between graphene layers, taking into account resulting bremsstrahlung radiation processes and their impact on the electrons' dynamics (details in Section S1, Supporting Information). We find that, at the considered kinetic energies, the electrons can still generate substantial X‐ray radiation through the interaction with the MRPs, as they can readily penetrate the entire structure even for device lengths of many dozens of microns (details in Section S1, Supporting Information). While the electron energy loss is small, it sets a limit to the performance of the device by limiting the distance the electrons can travel before experiencing significant energy loss and spread by scattering within the metamaterial. Electron energy loss and spread cause consequent emission spectra to shift and broaden, deteriorating the monochromaticity of each output radiation peak.

To analyze the collisions of electrons inside the device, we use the models proposed in refs. 49, 50, 51 and the Bethe formula for energy loss. For example, we find that for a metamaterial length *L* = 50 µm (as in Figure 1c), electrons with kinetic energy *T* = 5 MeV can easily traverse the entire device, the Lorentz relativistic factor γ being reduced by only about 0.4% (Section S1, Supporting Information, elaborates on the quantitative derivation and discussion). Nevertheless, electrons emit bremsstrahlung photons, representing a background on the output radiation. Such a background could be drastically reduced in a low‐density graphene metamaterial, for instance, employing a porous material as dielectric spacing. The bremsstrahlung could also be overcome with a very high MRP field (the radiation intensity scales as $E_{0}^{2}$),^4^ which in turn requires a high resilience of graphene and the entire metamaterial. Layered graphene structures could possibly improve the resilience of graphene beyond what has been achieved for a single layer. Indeed, it has been shown that encapsulating graphene with hexagonal boron nitride improves its quality and robustness.^29^, ^52^

We have shown that it is possible to excite MRPs of different orders in the graphene metamaterial by tuning the driving laser frequency. This allows us to directly shape the output X‐ray spectrum by controlling the incident laser. We have analyzed the role of the MRP orders *n* and *m* in the case of electrons travelling along the *z*‐direction. From Equation (1), we observe that output photons have higher energy for a larger MRP confinement factor (i.e., for a shorter period *p* or a higher MRP order *n*) and that the relevant parameter for the determination of the output energy is *p*/*n*. For instance, 8.5 keV photons in the forward direction from 5 MeV electrons can be obtained either for *p* = 33 nm and *n* = 1 or for *p* = 100 nm and *n* = 3 (Figure 2d). Higher order MRPs thus allow one to achieve higher output photon energies without decreasing the grating periodicity *p*.

The MRP momentum longitudinal and transverse components (with respect to the beam axis) can assume discrete values that depend on *n* and *m*. Thus, for a fixed beam direction, tuning the driving laser frequency makes it possible to vary the emission lines in frequency by discrete intervals. A scenario that is complementary to what we have considered in this paper is one where an electron beam is directed perpendicularly to the graphene sheets (i.e., parallel to *x*), for which the output energy would mainly depend on *m* and not on *n*. Such setup would take advantage of two factors: (1) the electron beam size can be increased as far as the device surface can be extended; (2) tuning the driving laser frequency, one will be able to (in principle) access MRPs of *N*_G_ different orders and, consequently, access *N*_G_ different emission energies. Alternatively, exciting multiple modes (*m*) would also enable emitting several lines at the same time. Furthermore, we note the possibility of *continuous tunability* of the energy and the emission spectral lines by simply changing the angle of incidence of the electron beam or of the driving laser. Altogether, by varying the geometrical and the material properties of the metamaterial and the grating, one can change the features of the MRPs and consequently of the output emission. For instance, an even larger tunability could be achieved with a nonuniform grating periodicity that generates higher spatial harmonics in the MRP spatial Fourier decomposition and, therefore, corresponding higher emitted EUV and X‐rays harmonics.

## 4 Conclusions

In conclusion, we presented the concept of using graphene metamaterials for plasmon‐driven free‐electron light sources. We considered a dielectric grating to couple the driving laser into the MRPs and used ab initio simulation tools to exactly model the physics of the laser–electron interaction. We showed the dependence of the X‐ray output intensity on the number *N*_G_ of graphene sheets in the metamaterial and highlighted the existence of an optimal number of graphene layers (*N*_G_^*^) maximizing the output radiation intensity. In this multilayer scenario, we showed that higher order MRPs generate higher output emission energy, circumventing the need of reducing the grating periodicity. We considered both a setup based on suspended graphene and a setup exploiting silica spacing layers (the latter being detailed in Section S1, Supporting Information), both of which share the same underlying physical mechanism for plasmon‐based X‐ray emission. We also discussed similar setups where the rotation of the electron beam or the employment of a grating with different periodicities could allow higher scalability and tunability of the light source. As an outlook, we note that preliminary studies suggest that similar devices could be realized exploiting other carbon structures such as carbon nanotubes.

Our study reveals a viable way of scaling up the output of nanophotonic free‐electron‐based radiative devices, paving the way to high‐yield compact tunable sources of EUV to X‐ray light, whose intensity can be readily scaled up by using electron beams of larger cross sections. With rapid advances in the development of few Hz narrow‐linewidth on‐chip lasers,^9^ graphene metamaterials are an optimal playground for the excitation of several variegated low‐losses high‐quality modes, opening the possibility to realize compact, dynamically tunable free‐electron light sources at extreme wavelengths.

## 5 Methods

Here, further details on the main steps of the simulations are provided.

*Exact Electromagnetic Field Solution*: The exact electromagnetic field solution, including the excitation of the MRPs, was obtained by means of 2D (dimensions *x* and *z*) finite‐element computations. Graphene was treated as a current boundary condition with Fermi energy *E*_F_ = 0.66 eV (i.e., scattering lifetime τ = 13.2 ps),^53^ electron mobility *µ* = 200 × 10^3^ cm^2^ V^−1^ s^−1^), and surface conductivity σ_g_ computed within the nonlocal random phase approximation (σ_g_ = σ_g_ (ω, *q*), with ω the driving laser central frequency and *q* = 2*πn*/*p* the longitudinal, that is, parallel to z, MRP wavevector, with *n* = 1, 2, … the MRP order).^14^ We choose the driving laser electric field strength *E*_0_ in such a way that the maximum field in the structure is *E*_max_ = 3 GV m^−1^ (see Section S3, Supporting Information, for further details). To avoid damaging the metamaterial, the considered *E*_max_ has to be smaller than the dielectric strength of the structure.^42^, ^54^

*Particle Tracking and Emitted Radiation Computation*: The Newton–Lorentz equation of motion was solved to find the electrons' trajectories through the MRP field with a Runge‐Kutta algorithm (for details, see the Supporting Information of refs. 4, 55). *N* = 200 particles in the bunch were simulated and the space charge is shown to be negligible, causing a divergence angle in the order of 10^−9^ rad.^4^ The emitted radiation is assumed to be incoherent, since spontaneous microbunching (self‐bunching) is negligible,^4^ as is shown in the simulations. Of course, given prebunched electrons, the radiation could become coherent. The exact radiation field was computed from the Liénard–Wiechert potentials.^45^ In presence of a dielectric spacing between the graphene layers, the electron–matter interaction by using standard tested techniques were accounted for.^56^ Specifically, the occurrence of scattering events for the electrons was treated stochastically and fictitious uniform electric fields were introduced to emulate the electron energy loss (details in Section S1, Supporting Information).

## Conflict of Interest

The authors declare no conflict of interest.

## Supporting information

Supplementary

Supplementary

Supplementary
