# Supplementary material for: Graphene Metamaterials for Intense, Tunable, and Compact Extreme Ultraviolet and X‐Ray Sources
Source: Adv Sci (Weinh). 2019 Oct 2;7(1):1901609. doi: 10.1002/advs.201901609 (PMC6947715; doi:10.1002/advs.201901609)
Supplement: Supplementary file 1 — Supplementary [file ADVS-7-1901609-s003.pdf]

Copyright WILEY-VCH Verlag GmbH & Co. KGaA, 69469 Weinheim, Germany, 2019.

ADVANCED SCIENCE
Open Access

Supporting Information

for *Adv. Sci.,* DOI: 10.1002/advs.201901609

Graphene Metamaterials for Intense, Tunable, and Compact Extreme Ultraviolet and X-Ray Sources

Andrea Pizzi, Gilles Rosolen, Liang Jie Wong, Rasmus Ischebeck, Marin Soljai, Thomas Feurer, and Ido Kaminer*

Supporting Information

Graphene metamaterials for intense, tunable and compact EUV and X-ray sources

Andrea Pizzi, Gilles Rosolen, Liang Jie Wong, Rasmus Ischebeck, Marin Soljačić, Thomas Feurer and Ido Kaminer*

S1: Dielectric spacing and electron-matter interaction

In this section, we consider an alternative design for the graphene metamaterial discussed in the main text that may be easier to realize. Instead of an array of suspended graphene sheets, we exploit a metamaterial where N_G graphene sheets alternate with $N_G - 1$ dielectric spacing layers. Such dielectric spacing layers could be, for example, a 2D heterostructure material like hexagonal boron nitride, or a 3D material like silica. This setup benefits from a higher mechanical stability and alleviates the requirements on vacuum system design, but would require electrons to pass through a solid target. While interacting with the MRPs, electrons will therefore scatter off the graphene-dielectric structure (Fig. S1a for the case of silica layers), generating phonons, bremsstrahlung photons, secondary electrons and positrons and possibly damaging the dielectric itself. For short enough structure lengths L such electron-matter interaction is negligible, but in general it is relevant for two main reasons. First, it directly results in the emission of bremsstrahlung radiation, that constitutes a background on the output radiation. Second, it perturbs the electron energies (i.e. velocities), affecting the radiation generated by the electron-MRP interaction. In this section we address these two issues by means of more complementary techniques, estimating the amount of bremsstrahlung radiation and the reduction of radiation from the electron-MRP interaction.

S1.1: Bethe approach for stopping power and bremsstrahlung

Electrons with high enough initial energy pass through the entire solid structure. However, electrons generally experience a progressive energy reduction due to the electron-matter interaction, which affects the radiation from the interaction with MRPs. To evaluate the average electron energy loss at a given penetration depth, we consider the semi-empirical formulae for the stopping power of a solid target based on the Bethe approach and provided by Seltzer *et al*. in ref. [1,2]. In Fig. S1b we report the stopping power of a solid Si target versus the electron energy, considering radiative and collisional contributions. In Fig. S1c we report the average relativistic Lorentz factor of the electrons $\gamma = \left(1 - \frac{v^2}{c^2}\right)^{-1/2}$ versus the penetration depth z into the solid Si as obtained integrating the stopping power of Fig. S1b for initial electron energies $T_0 = 10, 50, 100, 500, 1000, 5000$ keV. We observe that 5 MeV electrons experience an average energy loss of 0.04%, 0.20%, 0.41%, 0.82%, 1.22% at penetration depths $L = 5, 25, 50, 100, 150$ μm, respectively. For 5 MeV electrons and for the considered device lengths

($L \leq 150\ \mu m$), the stopping power is almost independent of the position, so that the emission of photons via bremsstrahlung (that depends on the energy as showed in Fig. S1b) occurs with a space-independent rate and the bremsstrahlung intensity grows linearly with L.

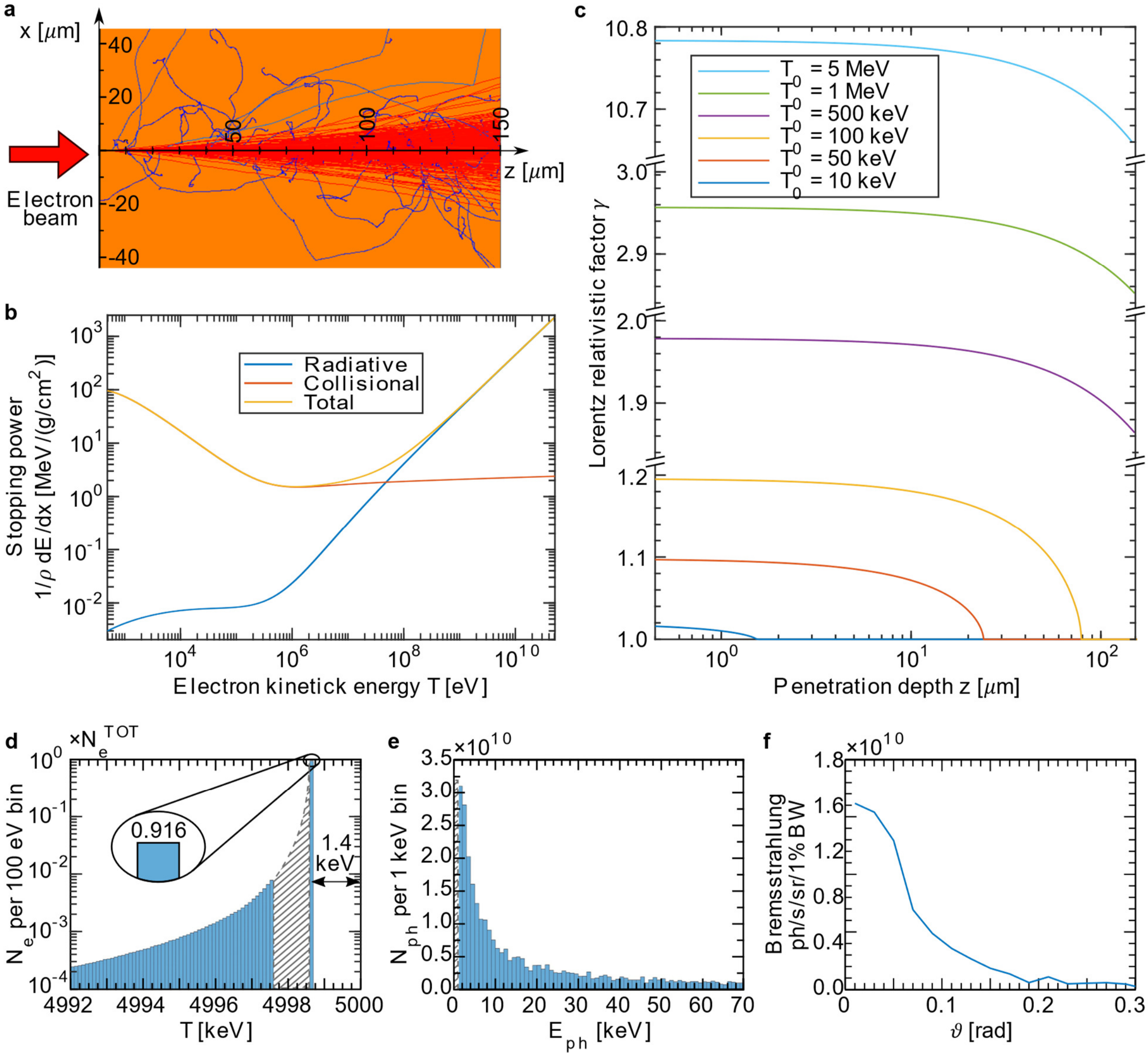

Figure S1 | Electron-matter interaction. (**a**) 5 MeV electrons' trajectories through a 150 µm long SiO_2 solid target obtained with Monte Carlo simulation software TRACER by GenISys for a beam of transverse $FWHM = 100$ nm and energy $FWHM = 1$ eV. Blue and red trajectories are for electrons that have and have not experienced catastrophic scattering, respectively. (**b**) Stopping power against electron kinetic energy T as obtained from ref. [1,2] for a Si target. (**c**) Electrons' relativistic factor γ against penetration depth in solid Si for different initial kinetic energies T_0 obtained numerically integrating the stopping power. (**d**,**e**) Histograms respectively of the output electron energy and of the bremsstrahlung photon energy, computed using the code EGSnrc (version 18770c) [3], for a SiO_2 target. In (**d**) we observe that, after 5 µm, 91.6% of the electrons are in the most energetic bin. The 1.4 keV loss of such bin with respect to the initial 5 MeV energy is attributed to sub-threshold collisions whereas the simulation does not capture points that fall within the shaded area in (**d**) due to the minimum modeled photon energy of 1 keV, reflected in the first empty bin in (**e**) (shaded column). (**f**) Intensity of the bremsstrahlung radiation at energy 6 keV against polar angle θ computed using the code EGSnrc.

S1.2: Bremsstrahlung radiation intensity

Quantifying the bremsstrahlung radiation is of fundamental importance, since it provides a background for the radiation generated by the electron-MRP interaction. To this end, we model the formation of the electromagnetic shower with the code EGSnrc [3], version 18770c. We run the code for a 10 pC bunch of electrons of kinetic energy 5 MeV and an interaction length $L = 5$ µm, exploiting the linearity of the bremsstrahlung intensity (at least for $L \leq 150$ µm, as explained above) to extend the results to a larger $L = 50$ µm, with a beam current $I_C = 40$ µA. A histogram of the number of emitted photons per 1 keV bin is shown in Fig. S1e. The minimum modeled photon energy in the code is 1 keV, which explains the first empty bin in the photon spectrum. In Fig. S1f we show the bremsstrahlung radiation intensity versus the polar angle θ at an emitted photon energy of 6 keV (bremsstrahlung radiation is isotropic with respect to the azimuthal angle ϕ). We find a maximum bremsstrahlung radiation intensity of 1.6×10^{10} ph/sr/s/1%BW, which compares favorably with the intensity of the radiation from electron-MRP interaction (Fig. S2b,c), of 7.85×10^{10} ph/sr/s/1%BW.

S1.3: Effects on the radiation from electron–MRP interaction

We now consider the effects of the electron energy loss and spread on the radiation from the electron-MRP interaction. In case of electrons interacting with MRPs in vacuum, such that all electrons have the same energy and travel in the same direction, the intensity of the radiation from the electron-MRP interaction scales as L^2 (L being the interaction length) whereas the width of the output spectral emission lines decreases as $1/L$ [4]. For the case of electrons interacting with the MRPs inside a solid target, electron energy loss and spread cause the intensity of the radiation to generally scale as L^ν with $\nu < 2$ and ν decreasing with L, corresponding to a broadening of the spectral emission lines. In order to maximize the ratio between the intensity of the radiation from the electron-MRP interaction and the bremsstrahlung radiation (that scales as L), we thus look for a tradeoff between increasing L and keeping ν appreciably above 1. For the graphene-silica metamaterial considered in Fig. S1 and 5 MeV electrons, we find an optimal structure length to be $L = 50\,\mu m$. To understand why, we elaborate a model that considers the interaction of the electrons both with the MRPs and with the solid device. In close analogy with the standard-tested techniques adopted in the code EGSnrc [3], we distinguish two classes of scattering events affecting the electrons: sub-threshold scattering events (STSE) and "catastrophic" scattering events (CSE).

Sub-threshold scattering events

This class of collisions contains the radiative and inelastic scatterings below certain energy thresholds k_c and T_c, respectively. STSEs are treated within a continuous slowing down approximation, that is introducing an effective electric field $\vec{E}_{st}(z) = E_{st}\hat{z}$. Therefore, for a given penetration depth the respective electron energy loss will be the *same* for *all* electrons. The highest bin of Fig. S1d (showing the histogram of electrons energies after 5 µm propagation inside the

solid target) allows us to isolate and quantify the energy loss due to STSEs, since it corresponds to the majority of electrons (e.g., 91.6% of the total) that experienced *only* STSEs.

Catastrophic scattering events

This class includes bremsstrahlung processes and other inelastic scattering events (such as the generation of secondary electrons) with energies above the thresholds k_c and T_c, respectively. Beyond reducing the average electron energy, CSEs induce an electron energy spread among electrons at a given penetration depth. Bremsstrahlung photons and secondary electrons are emitted over a wide spectrum, contributing to a broad range of energy losses and thus to energy spread. On the considered length and time scales, CSEs appear as discrete and stochastic, occurring on average once per length λ_c that an electron travels. The value of the mean scattering length λ_c is comparable with L. The first CSE for a bunch of electrons introduces a spread in their energies that significantly compromises their ability to radiate into a narrow and intense spectral emission line and is thus of crucial importance. It is therefore convenient to distinguish the radiation generated by the electrons before and after their first (possible) CSE. We accurately model the former (that contribute to the peak spectral emission line) and roughly model the latter (that is much less intense and generally emits outside the narrow spectral peak). At a given penetration depth, electrons that have only experienced the loss due to STSEs are monoenergetic. After travelling for a distance d, the first CSE occurs and electrons acquire a nonzero energy spread

$$\sigma_T(d) = \sqrt{1 + \frac{d}{\lambda_c}}\sigma_{T,1} \tag{S1}$$

with d/λ_c and $\sigma_{T,1}$ being the average number of CSEs after the first one and the energy spread after the occurrence of one CSE, respectively. In equation (S1), we assume that the electron energy spread after the occurrence of n CSEs is $\sigma_{T,n} \propto \sqrt{n}$ (corresponding to a diffusive process in the energy space). Additionally, we model the energy loss due to possible further CSEs after the first one with an effective field $\vec{E}_c(z) = \frac{\Delta_c}{e\lambda_c}\hat{z}$, e and Δ_c being the elementary charge and the average energy loss due to one CSE, respectively. Such a rough modelling of the electrons after their first CSE is reasonable since the main contribution to the output radiation comes from electrons which have not yet experienced any CSE.

Next, we explain how we use our *ab initio* simulation tool of the electron-MRP interaction in a way that includes the effects of both the STSEs and the CSEs. We divide the structure length L into K segments of length $l \ll \lambda_c$ (with l submultiple of L) and define $N_1^{(k)}$ the number of electrons experiencing a CSE for the first time in the k-th interval, with $k = 0, \dots, \mathrm{K} - 1$. We have

$$N_1^{(k)} \approx \frac{l}{\lambda_c} N_0(kl) \tag{S2}$$

with $N_0(z) = Ne^{-\frac{z}{\lambda_c}}$ the number of electrons that have not experienced any CSE at a distance z. The computation of the radiation from the electron-MRP interaction then distinguishes electrons before and after their first CSE. Assuming incoherent radiation, the total electron-MRP radiation

intensity is given by the sum of the intensities of the following $2K + 1$ computations, summarized in Table S1:

- One computation for the electrons that do not experience any CSE throughout the entire structure, considering a simulation length L, an initial energy $T = T_0 = 5$ MeV and the effective field $\vec{E}_{st}$ to mimic the continuous loss due to STSEs (row 1 of Table S1);
- One simulation for each k to consider $N_1^{(k)}$ electrons *before* their first CSE in the $(k+1)$-th segment, considering a simulation length $\left(k+\frac{1}{2}\right)l$, an initial energy $T = T_0$ and the effective field $\vec{E}_{st}$ (row 2 of Table S1).
- One simulation for each k to consider $N_1^{(k)}$ electrons *after* their first CSE in the $(k+1)$-th segment, considering a simulation length $L - \left(k+\frac{1}{2}\right)l$, an initial energy $T = T_0 - eE_{st}\left(k+\frac{1}{2}\right)l - \Delta_c$, the effective fields $\vec{E}_{st}$ and $\vec{E}_c$ to mimic the loss due to STSEs and CSEs, respectively, and a gaussian energy spread $\sigma_T\left(z = L - \left(k+\frac{1}{2}\right)l\right)$ to roughly take into account the spread due to the scattering events (row 3 of Table S1). The roughness of these K simulations is reasonable since their impact on the output emission intensity with respect to the other $K + 1$ simulations is very limited.

	Computation	Simulation length	Effective field	Initial energy	Gaussian energy spread
1	**No** CSEs	L	$\vec{E}_{st}$	T_0	0
2	**Before** first CSE, happening in the k-th segment	$\left(k+\frac{1}{2}\right)l$	$\vec{E}_{st}$	T_0	0
3	**After** first CSE, happening in the k-th segment	$L-\left(k+\frac{1}{2}\right)l$	$\vec{E}_{st} + \vec{E}_c$	$T_0 - eE_{st}\left(k+\frac{1}{2}\right)l - \Delta_c$	$\sqrt{1+\frac{L-\left(k+\frac{1}{2}\right)l}{\lambda_c}}\,\sigma_{T,1}$

Table S1. Computations of the radiation from electron-MRP interaction in a solid. Parameters of the $2K+1$ computations of the radiation from electron-MRP interaction when considering electron-matter interaction. One computation (row 1) accounts for the electrons experiencing no catastrophic scattering events (CSEs) inside the entire structure length L, K computations (row 2) consider electrons before their first CSE in the k-th segment and other K computations (row 3) consider electrons after their first CSE in the k-th segment ($k = 0,1,\dots,K-1$). The effective fields account for the electron average energy losses whereas the Gaussian energy spread roughly accounts for the spread due to CSEs.

Using the code EGSnrc [3], it is possible to obtain the parameters describing the electron-matter interaction. We run the code for 5 MeV electrons and $L = 5$ µm. As shown in Fig. S1d, we find that 91.6% of the electrons have experienced no CSE, from which we obtain the mean scattering

length $\lambda_c = -\frac{5\ \mu m}{\log 0.916} = 57$ µm, and that their energy loss per electron amounts to 1.4 keV, from which we get the effective STSEs friction field $E_{st} = \frac{1.4\ kV}{5\ \mu m} = 280\ \mathrm{MV/m}$. The remaining 8.4% of electrons can be assumed to have experienced only one CSE, since 5 µm $\ll \lambda_c$ and the probability of multiple CSEs is negligible. From these electrons we compute the energy standard deviation $\sigma_{T,1} = 61.5$ keV and mean loss $\Delta_c = 10.5$ keV, from which we find the effective field $E_c = \frac{\Delta_c}{e\lambda_c} = 180\ \mathrm{MV/m}$. For $L = 50\ \mu m$, the energy loss due to STSEs and CSEs amounts on average to $(E_{st} + E_c)eL = 23$ keV, which corresponds to a 0.46% electron energy loss.

S1.4: Summary of results

In Fig. S2, we present a selection of results obtained for the case of a graphene metamaterial with dielectric spacer material between the individual graphene sheets. Importantly, since the radiation from the electron interaction with MRPs scales as E_{max}^2 [4], it is possible to generate X-ray peaks that exceed the background bremsstrahlung radiation with an intense driving laser. In particular, we consider $E_{max} = 100\ \mathrm{GV/m}$ for the dielectric spacing-based setup. The breakdown threshold of the dielectric is above such field strengths [5], and the stability of graphene is expected to be increased by the layered structure. $N = 1000$ particles in the bunch are simulated, and details on the other parameters used for each figure are given in Table S2.

In Fig. S2a we show the electromagnetic absorption, from which we individuate a multitude of plasmonic modes that we label with the integers $n = 1,2, \ldots$ and $m = 0, 2, \ldots, N_G - 1$. We observe for small $q = 2\pi/p$ a hybridization of the modes, due to the overlap of branches with different n. Such phenomenon is not relevant for our study, which focuses on $p = 100$ nm, i.e. $2\pi\backslash p\backslash k_F \approx 0.062$. As the number of layers in the stack increases, the physical cross section increases but it becomes increasingly challenging for the driving laser to uniformly penetrate the stack, resulting in an optimum number of layers at which the output radiation is maximized (Fig. S2b). The output intensity is lower than in the case of suspended graphene-based setup since, as discussed in the previous sections, the electron-matter interaction causes an electron energy loss and spread and, therefore, a broadening of the output spectral emission lines (Fig. S2c).

As we increase the frequency of the driving laser, we excite higher order $n > 1$ MRPs, characterized by the electric field changing sign $2n$ times per period, as shown in Fig. S2f and Fig. S2g for $n = 2$ and $n = 4$, respectively. Of the infinity of modes present in the output spectrum, the n-th mode is the brightest one when the driving laser couples to the n-th order MRP, as shown in Fig. S2d and Fig. S2e for $n = 2$ and $n = 4$, respectively. The n-th pair of emission lines can be made even more dominant with respect to the others with a longer interaction length made possible by a longer structure.

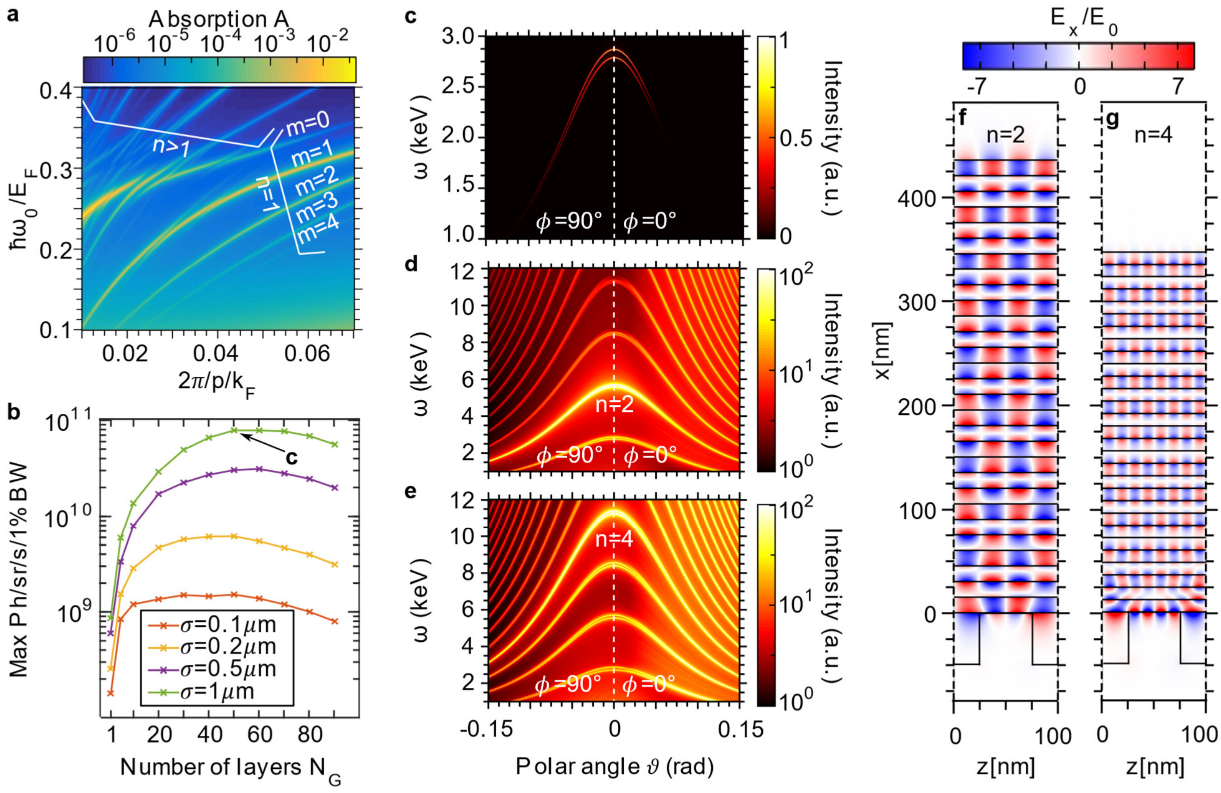

Figure S2 | Summary of results for a graphene-silica metamaterial. In analogy with Fig. 1, Fig. 2 and Fig. 3 of the main text, we report the main results for a SiO_2 spacing between the graphene sheets. The parameters of the computations are summarized in Table S2. (**a**) The electromagnetic absorption A as a function of the driving laser frequency ω_0 and grating periodicity p (k_F denotes the Fermi wavevector) exhibits multiple maxima corresponding to the dispersion of the MRPs. Notice that here we artificially reduced the electron mobility μ to increase the visibility of the absorption peaks, that would have been otherwise (that is for a larger μ) even narrower. (**b**) The output radiation intensity is maximized in the forward direction (i.e. at $\theta = 0°$) and can be increased with the number of layers in the stack. (**c**) The spectral angular distribution of the output radiation intensity $\frac{d^2I}{d\omega d\Omega}$ as a function of the frequency ω and the the polar angle θ at two fixed azimuthal angles $\phi = 0°$ (right) and $\phi = 90°$ (left), shows a pair of highly directional and nearly monochromatic spectral emission lines. (**d**,**e**) A pair of emission lines corresponds to each mode, but the brightest one is the one associated to the order n of the MRP, as highlighted adopting a logscale colorbar for $n = 2$ and $n = 4$, respectively. (**f**,**g**) Plotting the profile of the transverse electric field component E_x for one grating period we show MRPs of order n to be characterized by $2n$ lobes of the field per period in the $\boldsymbol{z}$ direction.

Figure	λ_0[μm]	N_G	L[μm]	p [nm]	s[nm]	(n, m)	σ[μm]	μ[cm^2/(Vs)]
Fig. S2a	var	5	-	var	25	var	-	5×10^3
Fig. S2b	var	var	50	100	25	(1, var)	var	200×10^3
Fig. S2c	7.086	50	50	100	25	(1,30)	1	200×10^3
Fig. S2d	4.921	30	5	100	15	(2, 5)	1	200×10^3
Fig. S2e	3.819	30	5	100	12	(4, 25)	1	200×10^3
Fig. S2f	4.921	30	-	100	15	(2, 5)	-	200×10^3
Fig. S2g	3.819	30	-	100	12	(4, 25)	-	200×10^3

Table S2. Computation parameters. Main parameters used in the computations of Fig. S2. We report the driving laser wavelength λ_0, number of graphene layers N_G, structure length L, grating periodicity p, dielectric spacing layers thickness s, MRPs' orders (n, m), electron beam transverse spread σ, graphene electron mobility μ and abbreviate *various* as *var*.

S2: Higher order plasmonic modes in graphene metamaterials

In the main text we showed that graphene metamaterials can support several higher order MRPs, that we label with two integers n and m. In this Supplementary Section we provide further details on such MRPs, showing their field profiles and how the order m impacts on the output radiation in the case of dielectric spacing-based setup. In Fig. S3a,b we show the field profile for a selection of higher order MRPs, each of them excited by a proper driving wavelength $\lambda_0(n, m)$, for a stack with $N_G = 10$ graphene sheets alternated with 9 SiO_2 spacing layers, a grating periodicity $p = 100$ nm, and a dielectric spacing thickness $s = 15$ nm. Each MRP corresponds to a different output emission spectrum and, as discussed in the main text, the position of the spectral emission lines for electrons travelling in the $\boldsymbol{z}$ direction is depending mainly on the order n. Here, we investigate the dependence of the output emission on m, showing (in Fig. S3c) the spectral angular distribution of the output radiation intensity corresponding to the fields of Fig. S3b, i.e. for $n = 1$ and $m = 3, 5, 7, 9$. We consider a structure with 100 periods (i.e. total length $L = 10\ \mu m$) with a driving field of $E_{max} = 100$ GV/m. The simulation includes $N = 1000$ electrons in the bunch, having a spread of $\sigma = 50$ nm. We see that, following equation (1), the central positions of the two spectral emission lines do not depend on m, whereas the distance between the two lines does. In fact, higher m MRPs are excited for lower frequencies and correspond thus to smaller distances between the two spectral emission lines of each pair. For large m, the two emission lines can eventually partially overlap and lead to an increase of the radiation intensity, as shown for $m = 9$ in Fig. S3c.

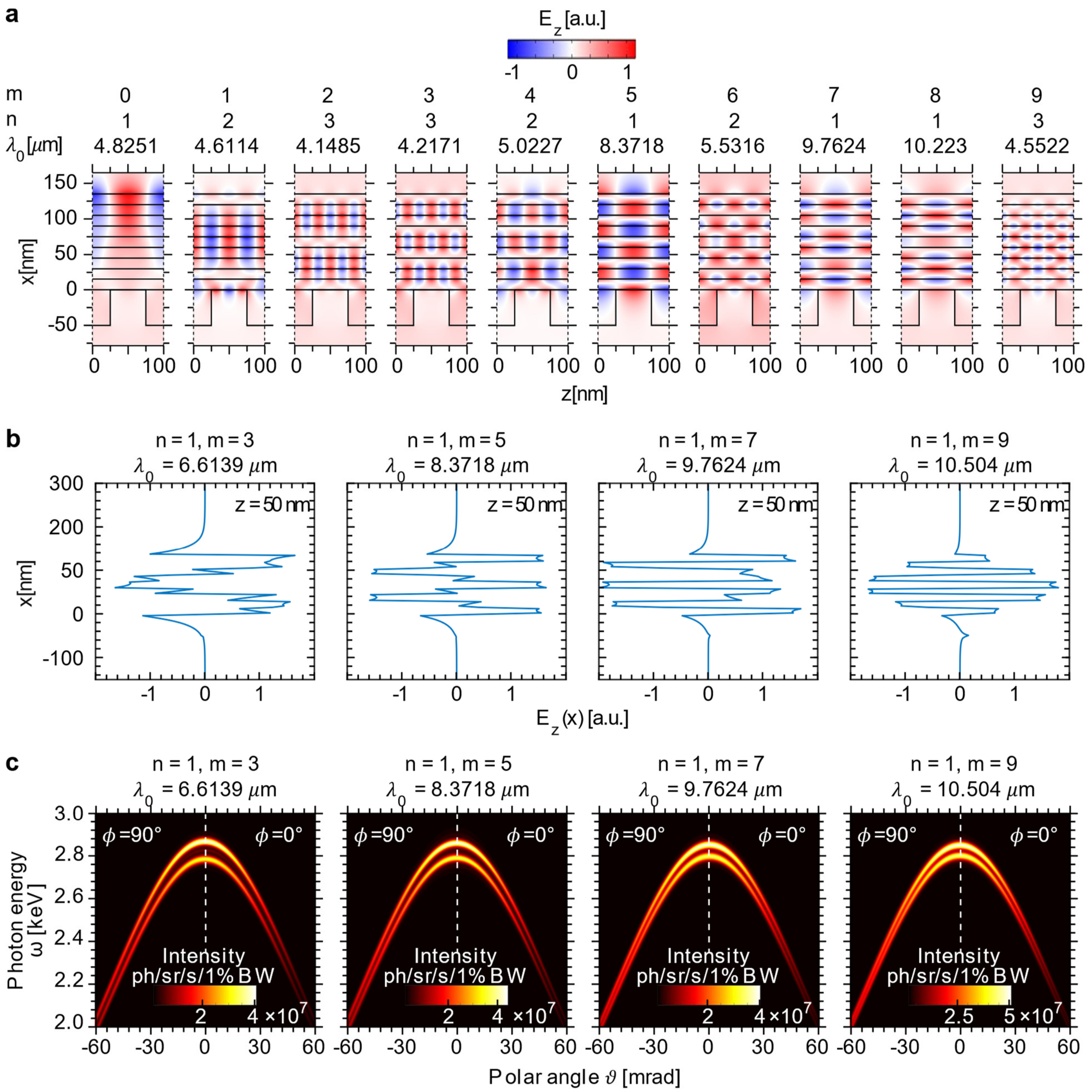

Figure S3 | Field profiles and light emission for higher order MRPs. We consider $N_G = 10$ layers in the stack. (**a,b**) Longitudinal component of the electric field for MRPs with various orders n, m, each one excited with the proper driving laser wavelength $\lambda_0(n,m)$ for a stack with spacing layers of thickness $s = 15$ nm and a grating periodicity $p = 100$ nm. We report here a representative selection of the MRPs with various $n = 1, 2, 3, \dots; m = 0, 1, 2, \dots, N_G - 1$. (**c**) Emitted radiation spectra corresponding to the MRP of (b) for an electron beam with spread $\sigma = 50$ nm and for a structure of length $L = 10$ μm (100 periods). In accordance with equation (2), we notice that a higher driving laser frequency (that is the smaller the order m) leads to a higher distance between the two spectral emission lines of a pair, whereas the position of the center of a pair is not affected by the order m of the MRP.

S3: Laser coupling efficiency and the increase of the driving laser intensity with the number of layers N_G

The coupling of the driving laser electric field strength E_0 to the graphene metamaterial depends on the exact design and results in a different maximum field E_{max} in each structure. The computations throughout the paper are done for a fixed maximum field in the structure E_{max}, that is reached by properly tuning the driving laser electric field strength E_0. For this discussion, we consider in fact the dielectric damage threshold as the limiting factor. In Fig. S4, we show the driving laser electric field strength E_0 versus N_G in the case of dielectric spacing between consecutive graphene sheets. The considered MRPs are of order $n = 1$ and $m \approx N_G/2$ and the parameters of the simulations correspond to the ones adopted in Fig. 1c. Not surprisingly, we find E_0 to increase with N_G, since a thicker stack supports more power before breaking. Note that determining the exact order m of a MRP for large N_G requires finding all the N_G MRPs with a given n and enumerating them. To avoid such a lengthy procedure, we restrict our attention to a MRP with frequency falling close to the center of the spectral range of the $n = 1$ MRPs and say it to be of order $m \approx N_G/2$.

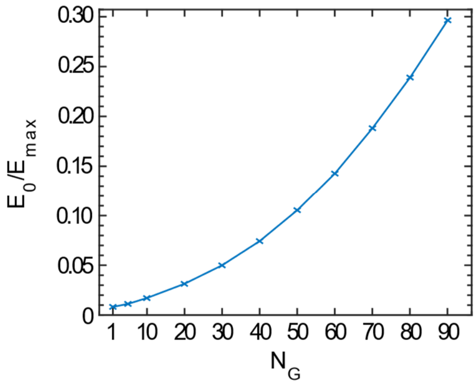

Figure S4 | Driving laser electric field strength E_0 for a different number of graphene layers N_G. For each N_G, we tune the driving frequency to a value ω_0 corresponding to a MRP of order $n = 1$ and $m \approx N_G/2$ (correspondingly to Fig. S2b) and look at the magnitude E_0 that produces a maximum field in the structure E_{max}. We observe an intuitive increasing trend, due both to the decrease of the absorption with the number of layers and to the fact that a larger N_G allows a distribution of the electromagnetic energy over a broader area.

S4: The effect of non-localities (spatial dispersion) in graphene

This section explains how the effect of graphene non-locality is considered in our formalism and simulations. Within the non-local Random Phase Approximation (RPA), the surface conductivity of graphene depends both on the frequency and on the momentum of the MRP, i.e. $\sigma_g = \sigma_g(\omega, q)$, with ω the driving laser central frequency and q the in-plane MRP wavevector (i.e. longitudinal, parallel to $\boldsymbol{z}$, from the point of view of the electrons). The mechanism that couples the driving laser to the MRPs in our graphene metamaterials is a grating that selects particular wavevectors, and therefore we can use the following approximation: we substitute $q = 2\pi n/p$ in σ_g as the MRP wavevector, with $n = 1, 2, 3, \ldots$ the order of the MRP [6]. To investigate

the effects of non-localities, we consider here a simpler setup, with a single graphene layer placed at a distance $t = 1$ nm above the grating, and with a driving laser incident at a perpendicular angle from below the substrate. In Fig. S5a,b we show the absorption A against the driving laser frequency ω_0 computed both within local RPA and non-local RPA, and we individuate peaks corresponding to plasmons of order $n = 1, 2, \dots, 9$. As the dispersion curve in Fig. S5c shows, the main effect of non-localities is to upshift the frequencies of the plasmons and, therefore, to increase the distance δω between the two spectral lines of the pairs in the output spectrum (equation (1)). For instance, for $p = 100$ nm and $n = 4$ (that is $q/K_F = 0.24$) we get a 2.5% increase of δω. Our analysis allows us to conclude that in our regime of interest the effect of non-localities is not dominant, which validates the approximations made in Fig. 3 and Fig. S2a.

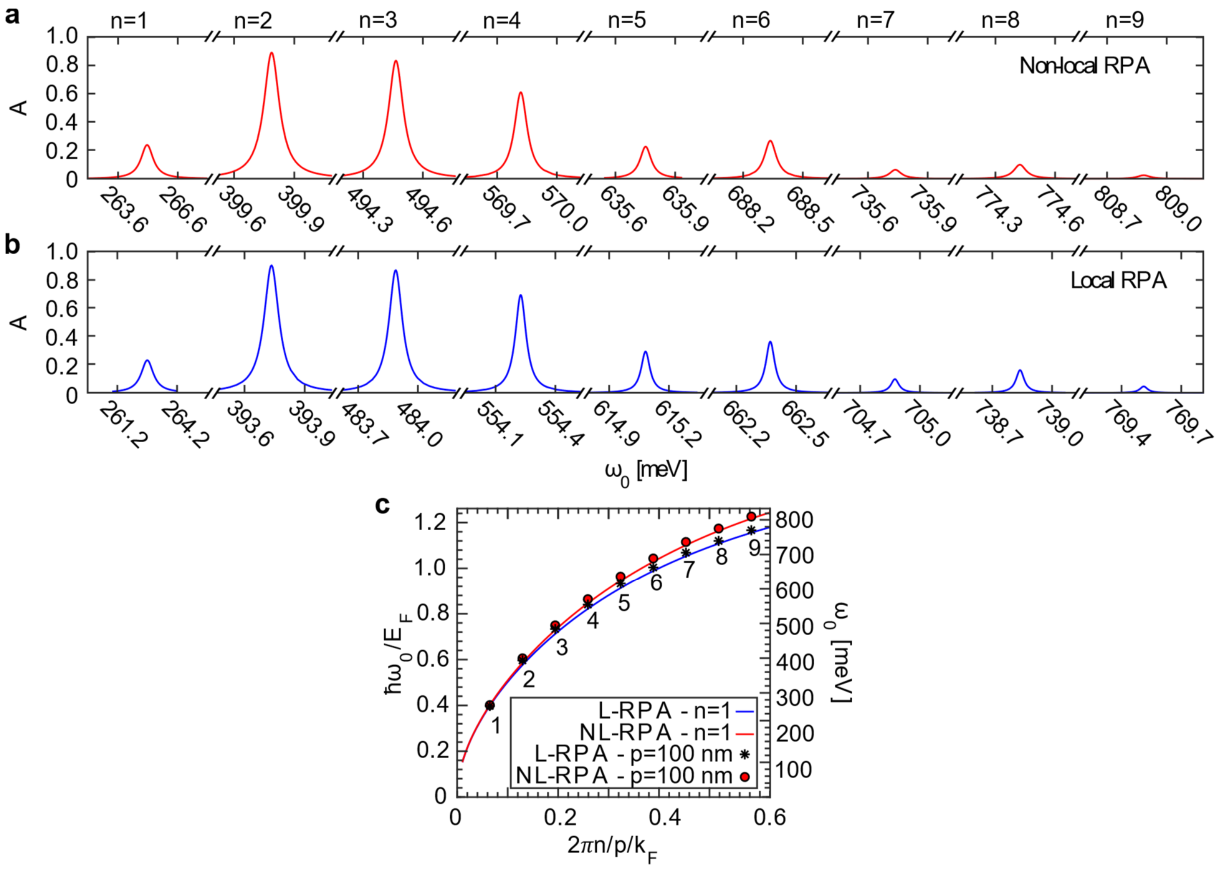

Figure S5 | Non-local effects in graphene plasmon properties: testing the changes of the plasmon dispersion relation and resonances in graphene. We compute the absorption A (**a**, **b**) and the dispersion curve (**c**) of the graphene plasmons both within local (L - blue line) and non-local (NL - red line) RPA for the very simple setup of a single graphene layer placed at a distance $t = 1$ nm above the grating and for a driving laser incident normal to the surface from below and polarized in $\boldsymbol{z}$. Each peak of the absorption corresponds to a different plasmon order n. For $p = 100$ nm, we observe that the main non-localities effect is to downshift the plasmon frequencies. For each grating periodicity p and plasmon order n, we identify the plasmon dispersion (**c**) with $\omega_p(q = 2\pi n/p/k_F) = argmax\ [A(\omega)]$. The numbers that label the solid circle markers in (**c**) indicate the plasmon order n for $p = 100$ nm. We observe how non-local effects are very small for the parameters considered in the present paper, that are $p = 100$ nm and $n = 1, 2, 4$ (corresponding to $2\pi n/p/k_F = 0.06, 0.012, 0.24$ respectively).

References

[1] Seltzer, Stephen M., and Martin J. Berger. "Procedure for calculating the radiation stopping power for electrons." *Int. J. Appl. Radiat. Isot*. **33**(11), 1219 (1982).

[2] Seltzer, Stephen M., and Martin J. Berger. "Improved procedure for calculating the collision stopping power of elements and compounds for electrons and positrons." *Int. J. Appl. Radiat. Isot*. **35**(7), 665 (1984).

[3] Kawrakow, Iwan. "Accurate condensed history Monte Carlo simulation of electron transport. I. EGSnrc, the new EGS4 version." *Med. Phys*. **27**(3), 485 (2000).

[4] Wong, L. J., *et al*. Towards graphene plasmon-based free-electron infrared to X-ray sources. *Nat. Photonics* **10**, 46 (2016).

[5] Berger, L. Dielectric strength of insulating materials. *Carbon* **1**, 2 (2006).

[6] Jablan, Marinko, Hrvoje Buljan, and Marin Soljačić. "Plasmonics in graphene at infrared frequencies." *Phys. Rev. B* **80**(24), 245435 (2009).
