# Supplementary material for: Graphene Metamaterials for Intense, Tunable, and Compact Extreme Ultraviolet and X‐Ray Sources
Source: Adv Sci (Weinh). 2019 Oct 2;7(1):1901609. doi: 10.1002/advs.201901609 (PMC6947715; doi:10.1002/advs.201901609)
Supplement: Supplementary file 3 — Supplementary [file ADVS-7-1901609-s002.docx]

**Supplementary Video title and caption.**

**Plasmon based X-ray sources**. Animated schematic of the device working principle. The driving laser couples to graphene plasmons (in red and blue) by means of a dielectric grating structure. The plasmonic field scatters the free-electrons (with trajectories as dashed black lines) into X-rays. (First part) In a single layer setup, only the electrons closest to the graphene contribute to X-rays generation. (Second part) A metamaterial supports instead plasmons with large transverse extension, thus offering a much larger cross section and leading to larger output power. (Third part) Higher order plasmons (e.g. $n=2$) wiggle electrons at higher frequencies, scattering them into higher X-rays harmonics.
